# Metabolic implications for predatory and parasitic bacterial lineages in activated sludge wastewater treatment systems

**DOI:** 10.1016/j.wroa.2023.100196

**Published:** 2023-08-13

**Authors:** Kyohei Kuroda, Shun Tomita, Hazuki Kurashita, Masashi Hatamoto, Takashi Yamaguchi, Tomoyuki Hori, Tomo Aoyagi, Yuya Sato, Tomohiro Inaba, Hiroshi Habe, Hideyuki Tamaki, Yoshihisa Hagihara, Tomohiro Tamura, Takashi Narihiro

**Affiliations:** a Bioproduction Research Institute, National Institute of Advanced Industrial Science and Technology (AIST), 2‐17‐2‐1 Tsukisamu‐Higashi, Toyohira‐Ku, Sapporo, Hokkaido 062‐8517 Japan; b Department of Science of Technology Innovation, Nagaoka University of Technology, 1603-1 Kamitomioka-Machi, Nagaoka, Niigata 940-2188 Japan; c Environmental Management Research Institute, National Institute of Advanced Industrial Science and Technology (AIST), 16–1, Onogawa, Tsukuba, Ibaraki 305–8569, Japan; d Bioproduction Research Institute, National Institute of Advanced Industrial Science and Technology (AIST), 1-1-1 Higashi, Tsukuba, Ibaraki 305-8566, Japan; e Biomedical Research Institute, National Institute of Advanced Industrial Science and Technology (AIST), 1-1-1 Higashi, Tsukuba, Ibaraki 305-8566, Japan

**Keywords:** Activated sludge, Metagenomics, Microbial community, Predatory bacteria, Symbiosis

## Abstract

Deciphering unclear microbial interactions is key to improving biological wastewater treatment processes. Microbial predation and parasitism in wastewater treatment ecosystems are unexplored survival strategies that have long been known and have recently attracted attention because these interspecies interactions may contribute to the reduction of excess sludge. Here, microbial community profiling of 600 activated sludge samples taken from six industrial and one municipal wastewater treatment processes (WWTPs) was conducted. To identify the shared lineages in the WWTPs, the shared microbial constituents were defined as the family level taxa that had ≥ 0.1% average relative abundance and detected in all processes. The microbial community analysis assigned 106 families as the shared microbial constituents in the WWTPs. Correlation analysis showed that 98 of the 106 shared families were significantly correlated with total carbon (TC) and/or total nitrogen (TN) concentrations, suggesting that they may contribute to wastewater remediation. Most possible predatory or parasitic bacteria belonging to the phyla Bdellovibrionota, Myxococcota, and *Candidatus* Patescibacteria were found to be the shared families and negatively correlated with TC/TN; thus, they were frequently present in the WWTPs and could be involved in the removal of carbon/nitrogen derived from cell components. Shotgun metagenome-resolved metabolic reconstructions indicated that gene homologs associated with predation or parasitism are conserved in the Bdellovibrionota, Myxococcota, and *Ca*. Patescibacteria genomes (*e.g.*, host interaction (hit) locus, Tad-like secretion complexes, and type IV pilus assembly proteins). This study provides insights into the complex microbial interactions potentially linked to the reduction of excess sludge biomass in these processes.

## Highlights

• Microbial community profiling of 600 activated sludges defined 106 shared families.
• High quality metagenome-assembled 1,184 bins were assigned to 97 shared families.
• Predatory Bdellovibrionota and Myxococcota may contribute to good water quality.
• Predation-related genes are widely conserved in Bdellovibrionales and *Myxococcaceae*.
• Homologues of symbiosis-related genes were identified in *Candidatus* Patescibacteria.

## Graphical abstract

**Figure:**
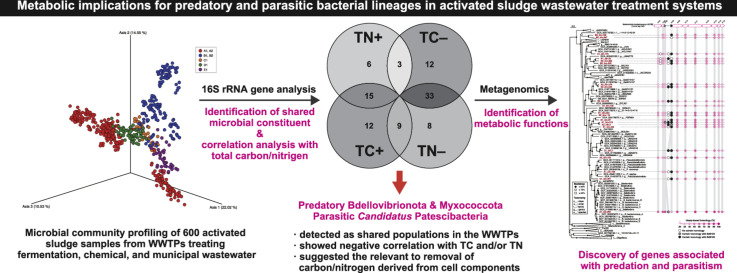

## Introduction

The activated sludge process has been used as a biological treatment technology for municipal sewage and industrial wastewater and has become a fundamental infrastructure in human society. Findings based on cultivation, microscopic observations, and molecular biological techniques, including Sanger sequencing and high-throughput sequencing, have shown that phylogenetically diverse arrays of microorganisms are involved in wastewater reclamation in activated sludge processes (Dueholm et al., 2022; Mei et al., 2017; Nielsen et al., 2004; Saunders et al., 2016; Wagner and Loy, 2002; J.K. Wu et al., 2019; Xia et al., 2018; Zhang et al., 2012; Zhang et al., 2023). In addition to microbial community profiling, shotgun metagenomic sequencing of activated sludge samples has been conducted to understand metabolic functions and microbial interactions within the activated sludge and the anaerobic digester microbiome (Liu et al., 2021; Nobu et al., 2020; Singleton et al., 2021; Ye et al., 2020). The essential microbiology of wastewater treatment processes (WWTPs) (*e.g.*, distribution of core microorganisms including phylogenetically novel microorganisms, and functional profiling in various activated sludge processes) is being established through leading-edge studies employing genetic information.

The next step in using these microbiological data is to identify the microbial populations that determine the efficiency of wastewater treatment from both economic and environmental perspectives, identify the environmental factors that affect these microbes, and elucidate the optimal operating conditions based on their metabolic functions. In the activated sludge process, oxygen, as an electron acceptor for respiration, is supplied by aeration to maintain the physiological activity of aerobic and facultative anaerobic microorganisms, resulting in vigorous cell growth and excess sludge. In certain cases, sewage and likely industrial WWTPs, excess sludge is treated by the methane fermentation process. Still, most of the rest is often dewatered and incinerated (Hara and Mino, 2008); thus, reducing excess sludge is a prerequisite for creating a sustainable society. Recently, the predatory and parasitic microorganisms have become the focus of attention to solve the unsolved excess sludge challenges facing the activated sludge process (L.W. Wu et al., 2019; Yu et al., 2017). Although the predatory effects of protozoa on WWTPs have been previously studied (Hao et al., 2011; Lee and Oleszkiewicz, 2003; Ni et al., 2011), advanced studies on predatory and parasitic prokaryotes that have not yet been isolated have been reported. Zhang *et al*. reported that members of the *Haliangium* and the uncultured mle1–27 clade of the phylum Myxococcota were identified as major predatory bacteria in municipal WWTPs using ^13^C rRNA-stable isotope probing (L. Zhang et al., 2023). In addition to these predatory bacteria, *Ca*. Patescibacteria are known to have episymbiotic or parasitic lifestyle with Actinobacteria (Batinovic et al., 2021; He et al., 2015), Gammaproteobacteria (Moreira et al., 2021; Yakimov et al., 2022), and methanogens (Chen et al., 2023, Kuroda et al., 2022a, Kuroda et al., 2022b), and may affect the carbon cycles in the activated sludge (Wang et al., 2023) and anammox processes (Hosokawa et al., 2021). In recent advanced finding revealed that *Ca.* Mycosynbacter amalyticus of the class *Ca*. Saccharimonadia were enriched from activated sludge obtained from municipal WWTPs using a co-cultivation technique and could lyse cells of *Gordonia amarae*, which is known as a causative agent of mycolata-associated foaming (Batinovic et al., 2021), suggesting the presence of unelucidated lytic ability against the host bacteria in WWTPs. However, there is still limited information about ecophysiology of predatory and parasitic bacteria in WWTPs because of the difficulty of the cultivation/isolation. In addition to the latest findings on predatory and parasitic organisms in municipal WWTPs, a metagenomic study of organisms in the process of treating various types of wastewater could provide a better understanding of activated sludge ecosystems that will lead to the development of technology for the reduction of excess sludge.

The study used 16S rRNA gene amplicon sequencing-based microbial community profiling to identify the shared microbial constituents, the family-level taxa commonly observed in 600 activated sludge samples from seven WWTPs treating industrial (fermentation and chemical processes) and municipal wastewater in Japan. The effects of water quality parameters, *i.e.*, total carbon (TC) and total nitrogen (TN) concentrations, on the shared microbial populations were estimated using statistical correlation analysis. Finally, shotgun metagenomic sequencing-based metabolic reconstruction was conducted to determine the ecophysiology and survival strategies of Bdellovibrionota, Myxococcus, and *Ca*. Patescibacteria as possible determinative microbial constituents of wastewater treatment ecosystems.

## Results and discussion

### Detection of the shared microbial constituents based on 16S rRNA gene profiling

**Fig. 1:**
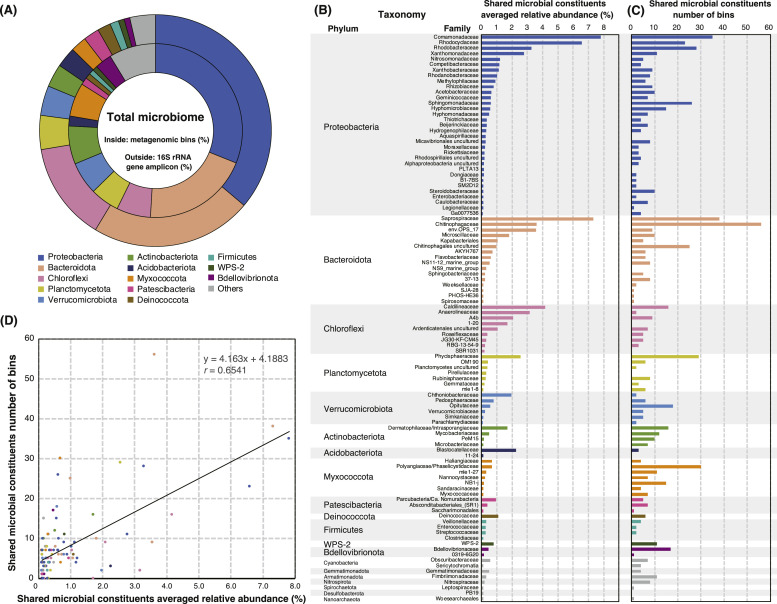
Overview of the microbial community dataset comprising of 600 activated sludge samples. (A) Abundances of microbial constituents at phylum level based on 16S rRNA gene amplicon sequencing (outside of pie chart) and shotgun metagenomic-assembled bins (inside). (B) The relative abundances of 106 shared microbial constituents are defined as “family-level taxa detected in all seven wastewater treatment processes (WWTPs) and have an average abundance of 0.1% or greater. (C) The number of metagenome-assembled bins assigned to the shared microbial constituents. (D) The relationship between the relative abundances of 16S rRNA gene-based microbial community profiling and the number of metagenome-assembled bins. The colors for bar charts (B and C) and scatter plots (D) correspond to those indicated in the pie chart (A).

The TC and TN concentrations and 16S rRNA gene-based microbial community profiles of the 600 sludge samples collected from the seven WWTPs treating fermentation (500 samples), chemical (61 samples), and municipal (39 samples) wastewater are shown in Table S1. A total of 41,584,738 reads from 16S rRNA gene amplicons were obtained, and 25,554 amplicon sequence variants (ASVs) with high Good's coverage values (>99%) were generated. ASVs associated with the phyla Proteobacteria, Bacteroidota, and Chloroflexi were predominant in the dataset, followed by Planctomycetota, Verrucomicrobiota, Actinobacteria, Acidobacteria, Myxococcota, *Ca*. Patescibacteria, Deinococcota, Firmicutes, WPS-2 (*Ca*. Eremiobacterota), and Bdellovibrionota (Fig. 1A, Table S2). Shotgun metagenomic sequencing provides approximately 17 G of high-quality reads from 72 representative samples (Table S1) and generates 1,184 high-quality (*i.e.*, ≥80% completeness) metagenome-assembled bins (Table S3) with a comprehensive gene annotation catalog by using DRAM software (Table S4). Reflecting that the TC and TN concentrations were not significantly different, principal coordinate analysis with weighted UniFrac showed that the 16S rRNA gene-based microbial communities of the municipal WWTP are not distantly related to those of the industrial WWTPs (Fig. S1). Thus, we attempted to detect the shared microbial components in a dataset comprising both industrial and municipal WWTPs. The 16S rRNA gene-based community profiling (Table S2) and metagenomic bins (Table S3) indicated that 51.9% and 72.1% were not classified to known genera, respectively (Fig. S2). Thus, we attempted to define the “shared microbial constituents” in this study as “family-level taxa that are detected in all seven WWTPs and have an average abundance of 0.1% or greater.” As a results, 106 families expanding 20 phyla representing 90.2% of the entire dataset were observed to meet this criterion (Fig. 1B, Table S5). This criterion is likely comparable to that is for core microbiome definition in MiDAS 3, although the resolution of taxa is different; *i.e.*, ASV-level taxa are observed in all WWTPs with an abundance of the top 80% of the dataset (Nierychlo et al., 2020). A total of 807 bins were assigned to 97 of the 106 shared families (Fig. 1C and Table S6). A positive linear correlation between the average relative abundance and the number of bins in the shared constituents was observed (Fig. 1D). These observations suggest that the dataset adequately estimates the diversity and metabolic functions of the activated sludge microbiome. The shared microbial constituents followed major microbial populations reported in previous studies on the microbial ecology of WWTPs (Dueholm et al., 2022; Nierychlo et al., 2020; Singleton et al., 2021), and their characteristics are described in the Supplementary Note.

### Correlations between the shared microbial constituents and TC/TN concentrations

**Fig. 2:**
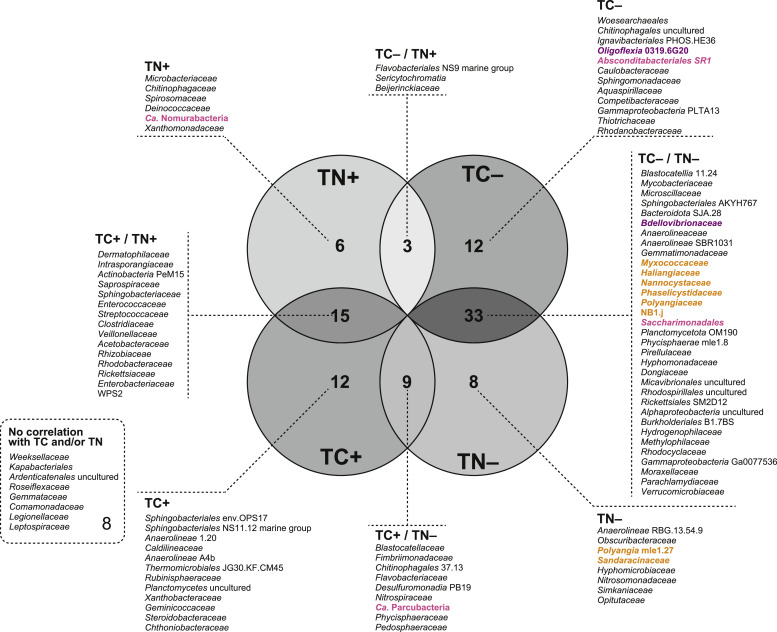
Venn diagram of the Spearman's rank correlations between the relative abundance of the shared microbial constituents, total carbon (TC), and total nitrogen (TN) concentrations of 600 activated sludge samples. TC+, shared families having a positive correlation with TC; TC−, those having a negative correlation with TC; TN+, those having a positive correlation with TN; TN−, those having a negative correlation with TN. The colors highlighted the populations of the phyla Bdellovibrionota (purple), Myxococcota (orange), and *Candidatus* Patescibacteria (magenta), which are identified as potential predatory or parasitic microbial constituents in the activated sludge samples.

Although previous investigations have identified core microbial constituents through large-scale microbial community analyses (Dueholm et al., 2022; J.K. Wu et al., 2019), information on the effects of fluctuations in water quality and operating conditions on population dynamics remains limited. One possible technical hurdle is the lack of consistent water-quality data obtained using a uniform analytical method corresponding to each activated sludge sample in the microbiome dataset. In this study, we acquired TC and TN concentration data for all 600 activated sludge samples collected (Table S1). Averaged values of TC concentration were 118.0 ± 45.6, 64.9 ± 12.4, 20.6 ± 4.9, 55.6 ± 25.5, 112.2 ± 39.2, 63.6 ± 33.6, and 47.1 ± 12.1; those of TN concentration were 44.5 ± 72.1, 356.1 ± 68.3, 7.7 ± 13.9, 61.1 ± 80.6, 31.7 ± 37.9, 9.1 ± 5.7, and 27.8 ± 21.2 in A1, A2, B1, B2, C1, D1, and E1 processes, respectively, indicating that carbon/nitrogen concentrations varied over a considerable range in each process. Statistical analysis based on Spearman's rank correlations between the relative abundances of TC and TN showed that 98 of 106 shared families had significant correlation(s) (*p* <0.005) with TC and/or TN (Fig. 2, Table S5), *i.e.*, 36 and 24 families were positively correlated with TC and TN, respectively, and 48 and 50 families were negatively correlated with TC and TN, respectively.

Interestingly, predatory and parasitic bacteria, which were classified primarily in the phyla Bdellovibrionota, Myxococcota, and *Ca*. Patescibacteria, were found to be the shared microbial constituents. Most of the shared families of these predatory bacteria were negatively correlated with TC and/or TN (Fig. 2), implying that a decrease in the available carbon and nitrogen components in wastewater may lead to an increase in predatory bacteria that can utilize cellular biomass as a nutrient. No clear correlations were observed between parasitic *Ca*. Patescibacteria, and TC/TN. Considering the limited range of host organisms available for *Ca*. Patescibacteria in wastewater streams (Albertsen et al., 2013; Batinovic et al., 2021), parasitic interactions may be governed by factors other than the carbon and nitrogen concentrations in the effluent. Because the populations of these predatory/parasitic bacteria were negatively correlated with phylogenetically diverse shared microbial families (data not shown), it was challenging to identify the microbes that were targets of predation and/or parasitism based solely on the results of the correlation analysis. Nevertheless, to clarify the metabolic functions of predation and parasitism in WWTPs, metagenome-informed metabolic reconstruction was conducted with particular emphasis on Bdellovibrionota, Myxococcota, and *Ca*. Patescibacteria.

### Potential predatory functions in Bdellovibrionota and Myxococcota

**Fig. 3:**
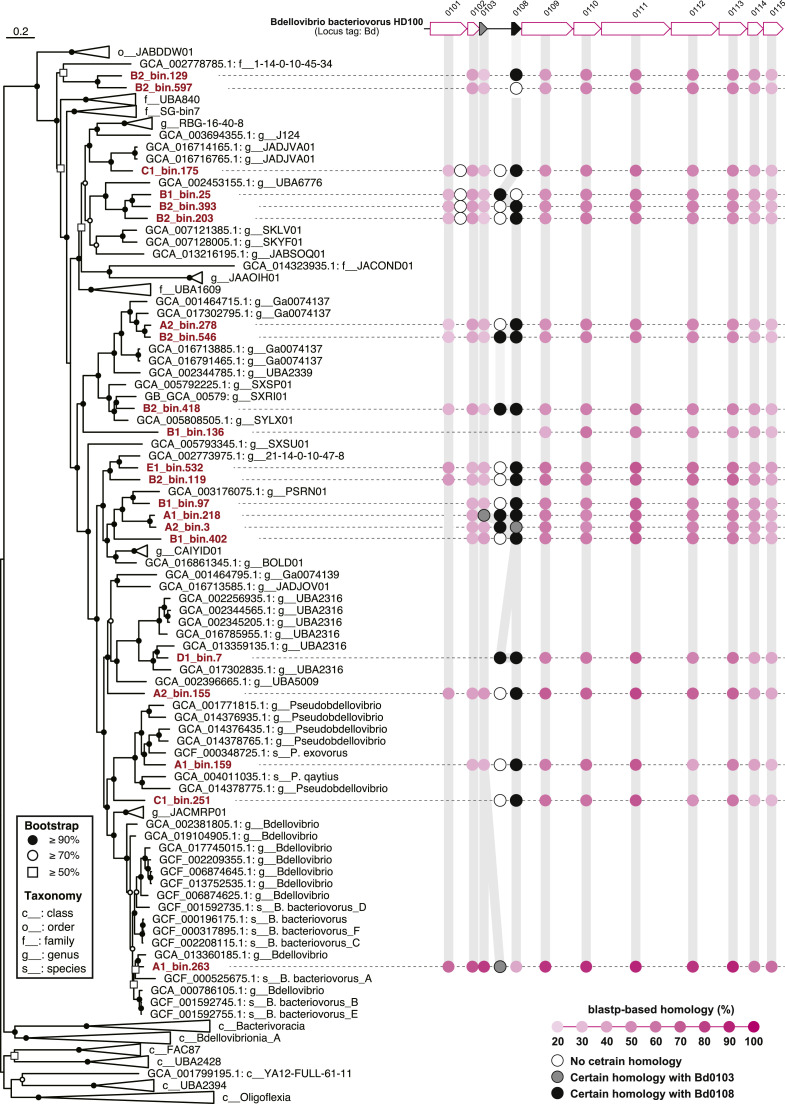
Genome tree of the phylum Bdellovibrionota based on concatenated phylogenetic marker genes in GTDBtk 2.0.0 (ver. r207) and pili and adherence gene clusters in metagenomic bins in the order Bdellovibrionales. Pink colored circles indicate a blastp-based homology (≥25% amino acid identity, ≤1e-5 e-value, and ≥50% query coverage per subject) with *Bdellovibrio bacteriovorus* HD100 (GCA_000196175.1). White, gray, and black circles are no certain homology with Bd0103 and Bd0108, certain homologies with Bd0103 (≤10 e-value), and certain homologies with Bd0108 (≤10 e-value), respectively.

Within the Bdellovibrionota and Myxococcota, only 8.1% (3 of 37 bins) and 18.0% (16 of 89 bins) of the metagenomic bins were classified at the genus level (Table S3). The environmental preference and genomic traits of these potential predators including known bacterial genera were described in Supplemental Note. To identify the potential predatory functions of the phylum Bdellovibrionota, a blastp-based homology search was performed against known pili and adherence gene clusters, including the host interaction (hit) locus of *B. bacteriovorus* HD100 (locus tag: Bd0108) (Rendulic et al., 2004). Twenty-one metagenomic bins of the phylum Bdellovibrionota possessed ≥14 genes out of 22 gene loci (≥60%). These bins belonged to *Bdellovibrionaceae* (14/17 bins), UBA1609 (4/5), and the unclassified families of order Bdellovibrionales (3/3) (Fig. 3 and Table S7), indicating that the gene cluster is widely conserved in the WWTP-associated Bdellovibrionales. The hit locus, which is an essential gene in host interactions such as type IV pilus formation and extension (Rendulic et al., 2004), was only identified in *Bdellovibrio*-related A1_bin.263 through relatively strict homology search conditions (≥25% amino acid identity, ≥ 1e-5 e-value, and ≥50% qcovs). Considering the possibility of underestimation due to the low e-value of the short amino acid length of the hit protein (102 aa), a blastp-based homology search was performed under more relaxed conditions (10 ≤ e*-*value). The results showed that 20 bins, except for B1_bin.136, had two or three short gene arrays (57–133 aa) close to the homologs of Bd0103 and Bd0109 of *B. bacteriovorus* HD100. In addition, the arrays contained genes with a certain homology (e-value: 1e-13–9.9) to the Bd0108 hit locus and signal peptides, suggesting that the gene arrays were secretory proteins similar to Bd0108 and Bd0103 (Rendulic et al., 2004) (Fig. 3 and Table S8). Further investigation is needed to clarify whether the gene arrays observed in the Bdellovibrionales bins function in host-predator interactions *in situ*.

**Fig. 4:**
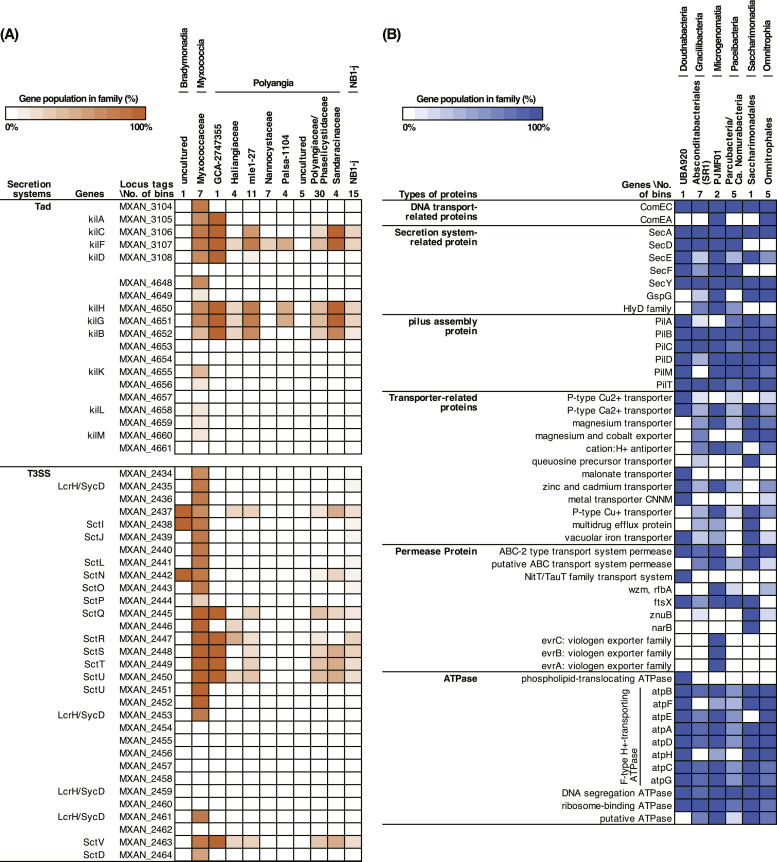
Summary of (A) possible cell contact-dependent predatory functions of the metagenomic bins of the phylum Myxococcota and (B) symbiosis-related genes and ATPase of the bins of the phylum *Candidatus* Patescibacteria/candidate phyla radiation (CPR) and *Ca*. Omnitrophota. (A) The tight adherence (Tad)-like systems and Type III secretion systems (T3SS) were annotated with the genome of *Myxococcus xanthus* DK1622 (GCA_000012685.1) at the thresholds of ≥25% amino acid identity, ≤1e-5 e-value, and ≥50% query coverage per subject. (B) The symbiosis-related genes and ATPase were annotated using the GhostKOALA pipeline (Altschul et al., 1990; Kanehisa et al., 2016). Gene populations were calculated based on the number of genes in each family. The detailed annotation results of (A) and (B) are listed in Tables S9 and S10, respectively.

To estimate the cell contact-dependent possible predatory functions of the bins of the phylum Myxococcota, secretion systems such as type III and tight adherence (Tad)-like systems, which are known to induce cell death and lysis (Thiery et al., 2022), were annotated by a blastp-based homology search with the genome of the *Myxococcus xanthus* DK1622 (Goldman et al., 2006). The results showed that homologs of the type VI secretion system (T6SS) are widely distributed in the bins of the class Polyangia except for the order Haliangiales, while the bins of class Myxococcia do not (Table S9). Bins of the class Bradymonadia and unclassified phylum-level taxon NB1-j (classified as class NB1-j of the phylum Myxococcota in the GTDB taxonomy) have limited genes related to secretion systems, indicating that cell contact-dependent predation mechanisms are quite different from *M. xanthus* DK1622. In the family *Myxococcaceae*, several homologs of Tad-like secretion complexes, named “Kil complex” and Type III secretion systems (T3SS) (Thiery et al., 2022), were identified in most of the bins (Fig. 4A and Table S9). Although a combination of motility, contact-dependent killing, outer membrane vesicles, and antibiotics is thought to be relevant for predation by *M. xanthus* (Thiery and Kaimer, 2020), recent studies have revealed that the effective digestion of prey cells is derived from contact-dependent killing via the Kil complex and T3SS (Seef et al., 2021; Thiery et al., 2022). These studies have shown that Tad and T3SS are required to induce cell death and degradation, respectively. Both secretion systems are essential for predation. Furthermore, predation experiments using *M. xanthus* mutant strains indicated that kilA, kilC, kilF, kilD, kilH, kilG, kilB, sctN, kilQ, and kilH are essential proteins for predation, whereas T3SS(2) is not involved in killing bacterial cells. In this study, most bins of the family *Myxococcaceae* possessed these important kil and sct genes in the Tad systems and the T3SS in their genomes, respectively (Fig. 4A and Table S9), suggesting that cell contact-dependent predation mechanisms are widely conserved in wastewater treatment-associated *Myxococcaceae*.

### Potential parasitism in wastewater *Ca*. Patescibacteria

*Ca*. Patescibacteria are known to grow symbiotically with their hosts in the environment (Castelle et al., 2018) and lack several essential biosynthetic pathways due to their small genome size (Parks et al., 2017). A total of 21 patescibacterial metagenomic bins obtained in this study have a small genome size (0.46 to 1.4 Mb) and lack most of the biosynthetic pathways necessary for cell growth, which are consistent with previous studies (Table S5 and Table S10) (Parks et al., 2017). Furthermore, the five bins were in the order of BD1–5 of class *Ca*. Gracilibacteria lacked most of the genes involved in the glycolysis pathway (≤ 33%). Previous studies reported poor metabolic pathways in the class *Ca*. Gracilibacteria (Fujii et al., 2022; Sieber et al., 2019). To further elucidate the lifestyle of *Ca*. Patescibacteria, we annotated symbiosis-related genes and ATPases in the recovered metagenomic bins (Fig. 4B and Table S10). In addition, we annotated these genes to the phylum *Ca*. Omnitrophota in a recent metagenomic study, in which most *Ca*. Omnitrophota bacteria are predators and parasites (Perez-Molphe-Montoya et al., 2022; Seymour et al., 2023).

Compared to *Ca*. Omnitrophota, the number of coding sequences (CDS) and the genome size of *Ca*. Patescibacteria was low (Tables S4 and S10) and the number of symbiosis-related genes in *Ca*. Patescibacterial bins were much smaller than those of *Ca*. Omnitrophota, indicating a strictly symbiotic lifestyle in wastewater treatment systems. Most of the metagenomic bins in *Ca*. Patescibacteria possess the DNA transport-related protein ComEC; secretion system-related SecADFY; type IV pilus assembly protein PilBCDT; cell division transport system permease protein ftsX; F-type *H*^+^/Na^+^-transporting ATPase; DNA segregation ATPase FtsK/SpoIIIE; and ribosome-binding ATPase (Fig. 4B and S10). For the unique genes in each class of *Ca*. Patescibacteria, homologs of the viologen exporter family transport system permease protein evrABC were observed only in the genome of the class *Ca*. Microgenomatia bins A2_bin.75 and A2_bin.201. The evrABC is known to function as a transporter of charged quaternary ammonium compounds (*e.g.*, methyl, ethyl, and benzyl viologen) that are essential for viologen extrusion (Prosecka et al., 2009). Although the detailed mechanism remains unclear, this unique exporter may extrude viologen-like compounds from *Ca*. Microgenomatia growth in the environment. Metagenomic bin C1_bin.50, belonging to the order *Ca*. Peribacterales (also known as order *Ca*. Absconditabacteriales in the SILVA taxonomy) of the class *Ca*. Gracilibacteria, only has the V/A-type ATPase, while the other *Ca*. Patescibacteria possess F-type ATPases. In addition, the branched-chain amino acid transport system, livHM, and lysine-specific permease were observed only in C1_bin.50, suggesting that external amino acids from the host and/or environment are required for growth. Among the class *Ca*. Paceibacteria (also known as *Ca*. Parcubacteria/OD1), have recently attracted much interest because of their parasitism of methanogenic archaea in anaerobic wastewater treatment sludge (Kuroda et al., 2022a, 2022b), a bin of the family *Ca*. Zambryskibacteraceae (B2_bin.460) harbors a gene cluster consisting of lactate permease, enolase, 2,3-bisphosphoglycerate-dependent phosphoglycerate mutase, d-lactate dehydrogenase, pyruvate-water dikinase, and glycerate 2-kinase. Since several studies have reported the presence of d- or l-lactate dehydrogenase in the *Ca*. Patescibacteria genomes (Chaudhari et al., 2021; Hosokawa et al., 2021), fermentative lactate production may be important for the growth of *Ca*. Zambryskibacteraceae. In short, the metagenomics-based metabolic reconstructions revealed that wastewater *Ca*. Patescibacteria may be symbiotic bacteria employing taxonomy-specific parasitic mechanisms along with the limited biosynthetic functions, which is consistent with a previous study (Wang et al., 2023).

## Conclusion

The study identified the shared microbial constituents of activated sludge WWTPs in Japan. Correlation analysis between 106 shared microbial populations and TC/TN parameters suggested that certain predatory or parasitic bacterial members of the phyla Bdellovibrionota and Myxococcota became dominant under low TC/TN conditions where wastewater treatment was stable. We created gene catalogs from 1,184 metagenomic-assembled bins, demonstrating the diverse metabolic functions of the shared microbes in activated sludge WWTPs. Furthermore, a detailed analysis employing this gene catalog led to the discovery of genes associated with predation and parasitism in Bdellovibrionota, Myxococcota, and *Ca*. Patescibacteria genomes, providing novel insights into the microbial interactions that may be relevant to the reduction of excess biomass. In the future, it will be essential to identify active microbial functions during on-site wastewater treatment using polyphasic approaches, including the cultivation/isolation of predatory/parasitic bacteria and transcriptome analysis. In addition, high-resolution microbiome analysis such as species level combining with RNA expression data, is required to link the exact microbial functions in activated sludge processes from the WWTPs. Ongoing efforts to develop technologies for reducing excess sludge based on microbial predation and parasitism mechanisms in activated sludge ecosystems will continue to be essential for a sustainable human society.

## Materials and methods

### Sludge sampling

Activated sludge samples were collected from seven full-scale wastewater treatment processes (WWTPs) treating fermentation wastewater (sites A1, A2, B1, B2, and C1), chemical wastewater (site D1), and municipal wastewater (site E1) in Japan. All wastewaters did not contain specific organic carbon or nitrogen species and/or toxic chemicals. After centrifugation (8,500 × *g,* 10 min), the precipitated sludge and supernatant were collected separately. The supernatant samples were filtered with a 0.22 µm pore-sized membrane filter (Millex-GV Syringe Filter Unit, 0.22 µm, PVDF, 33 mm, Merck, Rahway, NJ, USA). The samples were stored in an −80 °C freezer before use for DNA extraction and chemical analyses. In this study, due to technical limitations, we have measured only TC and TN concentrations, which are hypothetically considered to affect wastewater treatment performance. TC and TN concentrations were analyzed using a TOC-TN analyzer (TOC-L/TNM-L; Shimadzu, Kyoto, Japan) (Inaba et al., 2018).

### DNA extraction and 16S rRNA gene-based microbial community profiling

Total DNA was extracted from the sludge samples using a direct lysis protocol that included bead beating, phenol-chloroform extraction, and ethanol precipitation (Yamada et al., 2005). PCR amplification using the universal primer sets Univ515F (5′-GTGCCAGCMGCCGCGGTAA-3′) and Univ806R (5′- GGACTACHVGGGTWTCTAAT-3′) (Caporaso et al., 2012) was performed as previously described (Kuroda et al., 2021). 16S rRNA gene amplicon sequencing was performed using the MiSeq Reagent v2 kit (Illumina, San Diego, CA, USA) as previously described. Raw paired-end sequences were joined using the fastq-join tool in the eautils software package (version 1.3.1) (Aronesty, 2011). The joined sequence data were quality-filtered by deblur using a sequence length (≥250 nt) and quality score (≥30) cut-off (Amir et al., 2017) using the QIIME2 platform (version 2021.11) (Bolyen et al., 2019). The amplicon sequence variants (ASVs) obtained were aligned with MAFFT (Katoh et al., 2002) and used to construct a phylogeny using fasttree2 (Price et al., 2010). Alpha diversity metrics were calculated, including Chao1 (Chao, 1987) and Good's coverage (Good, 1953). Taxonomy was assigned to each ASV using the q2‐feature‐classifier (Bokulich et al., 2018) with a classify‐sklearn naïve Bayes taxonomy classifier against the SILVA 138 reference sequences (Quast et al., 2013). Beta diversity metrics with weighted UniFrac (Lozupone et al., 2007) and principal coordinate analysis (PCoA) were estimated using q2‐diversity after samples were rarefied (subsampled without replacement) to 6,000 sequences per sample. In this study, “shared microbiomes” were defined as those detected in all seven processes included in the analysis and having an average relative abundance of 0.1% or greater. Spearman's rank correlation coefficients (*rs*) between the shared microbial constituents and chemical parameters (TC and TN concentrations) were calculated using R software (R Core Team, 2018).

### Metagenomic shotgun sequencing, assembly, and binning

Metagenomic shotgun sequencing and data analysis were performed as previously described (Kuroda et al., 2021). Briefly, the extracted DNA was used for shotgun sequencing to generate high-accuracy reads in 2 × 150 mode on a NovaSeq sequencer (Illumina, San Diego, CA, USA). The obtained reads were trimmed using Trimmomatic v0.33 (Bolger et al., 2014) and assembled using MEGAHIT v.1.2.9 (Li et al., 2015). Assembled reads were binned using MetaBAT v.2.12.1 (Kang et al., 2015). The completeness and contamination of each bin were checked using Check M v.1.0.11 (Parks et al., 2015). High-quality metagenome-assembled bins (≥80% completeness and <10% contamination) were selected for subsequent analyses. The bins of phylum *Ca.* Patescibacteria were chosen at thresholds of ≥60% completeness and <10% contamination because of the limited number of marker genes in their genomes (Parks et al., 2017). The taxonomic classification of each bin was estimated using GTDBtk v.1.4.1 (GTDB release95; default parameters) (Parks et al., 2018). Short contigs (<2,000 bp) were removed. Genes were annotated using a combination of KEGG (Ogata et al., 1999), GhostKOALA (Altschul et al., 1990; Kanehisa et al., 2016), IMG/M (Markowitz et al., 2014), Prokka (Seemann, 2014), DRAM (Shaffer et al., 2020), and manual annotations. For annotations of a pili and adherence gene cluster of the phylum Bdellovibrionota (Rendulic et al., 2004), phylogenetically associated bins were compared to the genome of *Bdellovibrio bacteriovorus* HD100 (GCA_000196175.1) at a thresholds of ≥25% amino acid identity, ≤1e-5 e-value, and ≥50% query coverage per subject (qcovs). To further annotate the host-interaction (hit) locus in the gene cluster, a blastp-based homology search with a known gene (locus tag: Bd0108) and a neighboring small gene (Bd0103) of *B. bacteriovorus* HD100 was performed with the threshold of ≤10 e-value. The signal peptides of the gene clusters were annotated using SignalP 6.0 (Teufel et al., 2022). A genomic tree was constructed using concatenated phylogenetic marker genes from the obtained bins and genomes of the order Bdellovibrionales. Conserved marker genes were identified using “gtdbtk identify” with default parameters and aligned to reference genomes using “gtdbtk align” with taxonomic filters (–taxa_filter p__Bdellovibrionota) using GTDBtk v2.0.0 (GTDB release207; default parameters) (Chaumeil et al., 2020). A phylogenetic tree of the bins was constructed using IQ-TREE version 2.1.4-beta (-B 1000) with an automatically optimized substitution model (Q. yeast +R10) (Minh et al., 2020). The potential predatory functions of the metagenomic bins of the phylum Myxococcota were estimated using blastp ver. 2.6.0 with known genes of secretion systems relevant to bacterial predation in the genome of *Myxococcus xanthus* DK1622 (GCA_000012685.1), at a threshold of ≥25% amino acid identity, ≥1e-5 e-value, and ≥50% qcovs (Thiery et al., 2022).

### Nucleotide sequence accession number

The sequence data obtained in this study were deposited into the DDBJ Sequence Read Archive (SRA) database under accession numbers DRA015582 and DRA016086, which were used for shotgun metagenomics and 16S rRNA amplicon sequencing, respectively.

## Data availability

Data will be made available on request.

## Appendix. Supplementary Materials

Supplementary Notes. “The characterization of the shared microbial constituents based on current knowledge of wastewater treatment microbiology” and “The ecological information on shared genomes within industrial and municipal WWTPs.”

Fig. S1. Weighted UniFrac-based principal coordinate analysis (PCoA) plot of 600 activated sludge samples.

Fig. S2. Percentage of the assigned (gray) and unassigned (white) microbial populations at genus level taxonomy for the shared microbial constituents in (A) community profiling by 16S rRNA gene amplicon sequencing and (B) metagenomic bins. N.D., not detected.

Table S1. Sample information

Table S2. Relative abundances of microbial community assemblages at the genus level

Table S3. Detailed information on metagenome-assembled bins

Table S4. Comprehensive gene annotation of metagenome-assembled bins by using DRAM software

Table S5. Relative abundances and correlation with the shared microbial constituents’ total carbon and nitrogen parameters

Table S6. Metagenome-assembled genomes for the shared microbial constituents

Table S7. The host interaction gene locus of Bdellovibrionota-related bins

Table S8. The host interaction gene locus with relatively low similarity among Bdellovibrionota-related bins

Table S9. The predation-associated gene locus of Myxococcota-related bins

Table S10. The symbiosis-associated genes in *Candidatus* Patescibacteria-related bins

## Declaration of Competing Interest

The authors declare that they have no competing financial interests or personal relationships that may have influenced the work reported in this study.

## Data Availability

Data will be made available on request.

## Appendix Supplementary materials

Image, application 1

Image, application 2

Image, application 3

Image, application 4
