# Supplementary figures and images for: Metabolic implications for predatory and parasitic bacterial lineages in activated sludge wastewater treatment systems
Source: Water Res X. 2023 Aug 13;20:100196. doi: 10.1016/j.wroa.2023.100196 (PMC10469934; doi:10.1016/j.wroa.2023.100196)

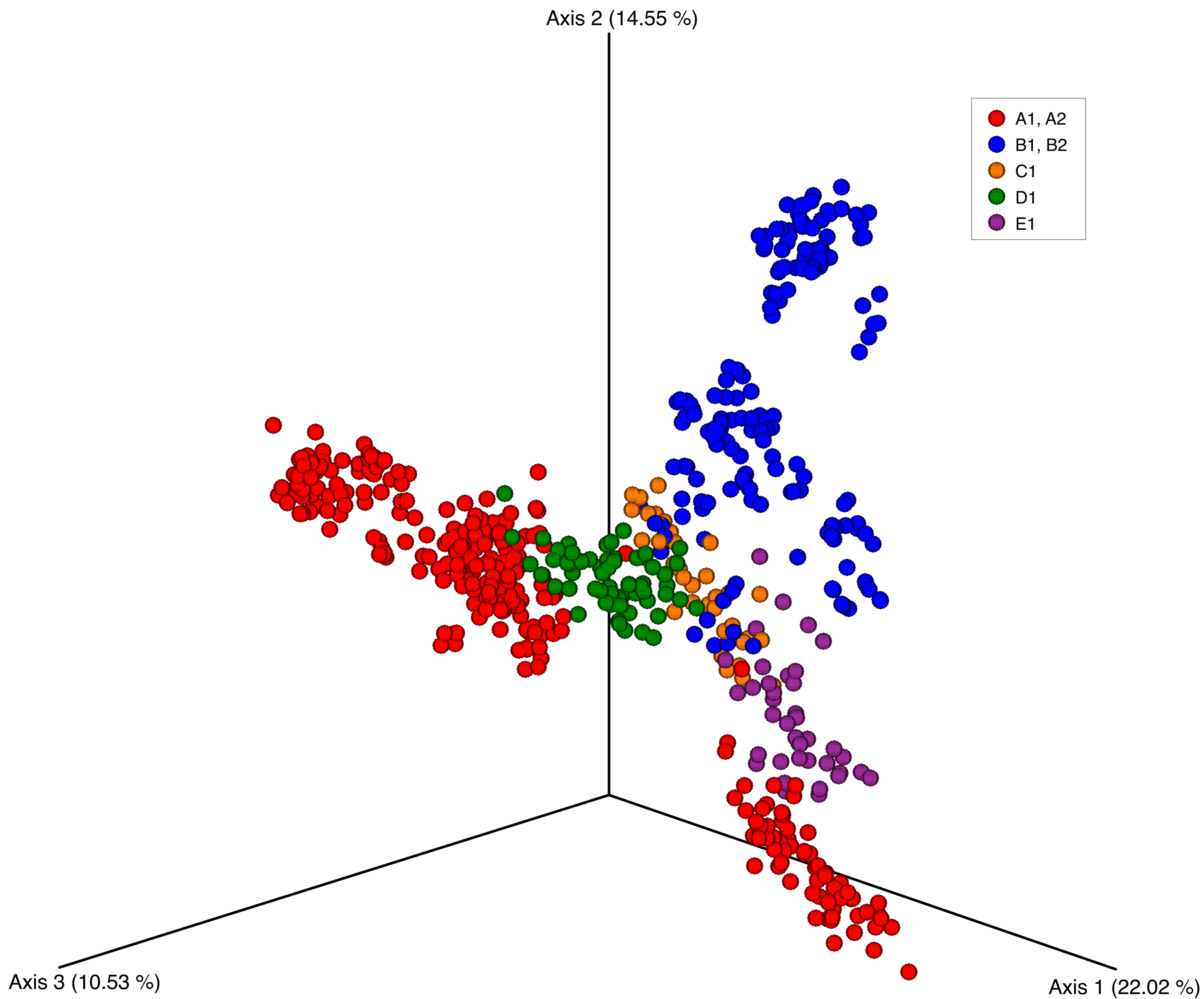

**Fig. S1**

Supplement: Supplementary file 1 [file mmc1.pdf]

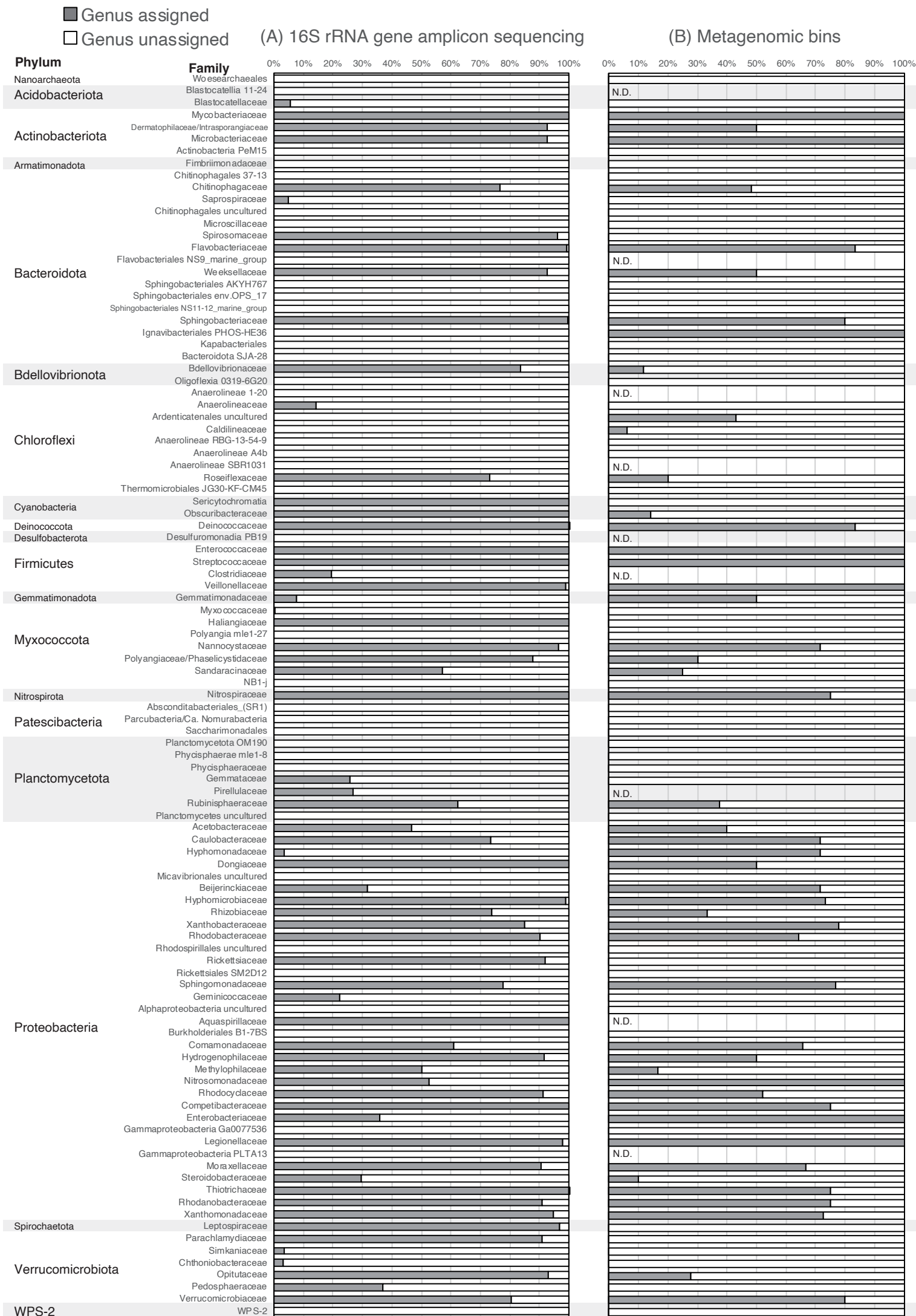

Supplement: Supplementary file 2 [file mmc2.pdf]
