# Supplementary material for: Metabolic implications for predatory and parasitic bacterial lineages in activated sludge wastewater treatment systems
Source: Water Res X. 2023 Aug 13;20:100196. doi: 10.1016/j.wroa.2023.100196 (PMC10469934; doi:10.1016/j.wroa.2023.100196)
Supplement: Supplementary file 3 [file mmc3.pdf]

– Supporting Information–

Metabolic implications for predatory and parasitic bacterial lineages in activated sludge wastewater treatment systems

Kyohei Kuroda [1*], Shun Tomita [1], Hazuki Kurashita [1, 2], Masashi Hatamoto [2], Takashi Yamaguchi [2], Tomoyuki Hori [3], Tomo Aoyagi [3], Yuya Sato [3], Tomohiro Inaba [3], Hiroshi Habe [3], Hideyuki Tamaki [4], Yoshihisa Hagihara [5], Tomohiro Tamura [1], Takashi Narihiro [1*]

[1]Bioproduction Research Institute, National Institute of Advanced Industrial Science and Technology (AIST), 2-17-2-1 Tsukisamu-Higashi, Toyohira-ku, Sapporo, Hokkaido, 062-8517 Japan

[2] Department of science of technology innovation, Nagaoka University of Technology, 1603-1 Kamitomioka-machi, Nagaoka, Niigata, 940-2188 Japan

[3] Environmental Management Research Institute, National Institute of Advanced Industrial Science and Technology (AIST), 16–1, Onogawa, Tsukuba, Ibaraki 305–8569, Japan

[4] Biomedical Research Institute, National Institute of Advanced Industrial Science and Technology (AIST), 1-1-1 Higashi, Tsukuba, Ibaraki 305-8566, Japan

[5] Bioproduction Research Institute, National Institute of Advanced Industrial Science and Technology (AIST), 1-1-1 Higashi, Tsukuba, Ibaraki 305-8566, Japan

[*]Co-corresponding authors.

Kyohei Kuroda, Tel: +81 11 857 8402; E-mail: k.kuroda@aist.go.jp

Takashi Narihiro, Tel: +81 29 861 9443; E-mail: t.narihiro@aist.go.jp

<u>Supplementary Notes</u>

The shared microbial constituents followed major microbial populations reported in previous studies on the large-scale microbial community analyses of WWTPs. As one of the remarkable initiatives, the Microbial Database for Activated Sludge (MiDAS) platform consists of 16S rRNA gene amplicon-based microbial community data from 740 wastewater treatment processes (WWTPs) in 31 countries and provides a global view of common microbial constituents in wastewater treatment ecosystems with full-length 16S rRNA genes as highly accurate references for the comprehensive taxonomy from domain to species level (Dueholm et al., 2022; Nierychlo et al., 2020). The ecological features of shared microbial constituents identified by our dataset are described as follows.

The characterization of the shared microbial constituents observed in this study based on current knowledge of wastewater treatment microbiology

Within the shared microbial constituents of Proteobacteria, it was observed to consist of the *Comamonadaceae* (7.82 %) and *Rhodocyclaceae* (6.58 %), including denitrifying bacteria such as the genera *Comamonas*, *Thauera*, and *Zoogloea*, as the major microbial constituents. Ammonia-oxidizing *Nitrosomonadaceae* (1.20 %) and nitrite-oxidizing *Nitrospiraceae* (0.21 %) of the phylum Nitrospirota were detected as shared microbial constituents, suggesting that these N-cycling bacteria are responsible for nitrogen removal in WWTPs. According to the correlation analysis between the relative abundance of the shared microbial constituents and TC and TN concentrations, the populations of these N-cycling bacteria and methanol-utilizing *Methylophilaceae* were negatively correlated with TN and TC, respectively, indicating that they may be abundant under good nitrogen/carbon removal conditions. In addition, members of *Ca*. Accumulibacter of the *Rhodocyclaceae* and *Ca*. Competibacter of the *Competibacteraceae* family were detected as the shared microbial constituents associated with the competition for phosphate removal.

To date, several bacterial lineages are known to possess the predation traits including the members of the Bacteroidota (e.g., *Saprospira*), Bdellovibrionota (e.g., *Bdellovibrio* and *Micavibrio*), Myxococcota (e.g., *Myxococcus* and *Sorangium*) and Chloroflexi (e.g., *Herpetosiphon*) (Pasternak et al., 2013). Predatory bacteria of the genus *Micavibrio* have been classified into the family *Micavibrionaceae*, order Micavibrionales, and class Alphaproteobacteria (Davidov et al., 2006; Lambina et al., 1982). In this study,

although it has been reclassified into the family *Pseudobdellovibrionaceae* of the order Bdellovibrionales (Hahn et al., 2017; Waite et al., 2020), it is displayed as a member of Alphaproteobacteria according to the SILVA (Quast et al., 2013), GTDB (Chaumeil et al., 2020), and MiDAS (Dueholm et al., 2022) classifications. Since the dominant amplicon sequence variants (ASVs) of Micavibrionales detected as the shared microbial constituents showed relatively low (<90 %) 16S rRNA gene identity to known bacterial isolates on BLASTN analysis (data not shown) (Camacho et al., 2009), it is unclear whether the Micavibrionales members possess predation traits. Two families, Rickettsiaceae and an unidentified clade, SM2D12 (known as UBA998 in the GTDB taxonomy), were detected in the shared microbial constituents of Rickettsiales. Within the family Rickettsiaceae, ASVs were associated with *Ca*. Megaira, known to be symbiotic bacteria of algae and ciliates (Davison et al., 2023; Schrallhammer et al., 2013), and were predominant (Table S3). In addition, the metagenome-assembled genomes of SM2D12/UBA998-related bacteria have been observed in aquatic environments and may possess free-living lifestyles (Moncadas et al., 2023). These findings suggest unknown interactions between eukaryotes such as algae and ciliates and uncultured Rickettsiales bacteria in wastewater ecosystems may occur. These potential predatory/parasitic bacterial populations (i.e., the uncultured family of Micavibrionales and Rickettsiales) were negatively correlated with TC and TN (Fig. 2), suggesting their proliferation under limited carbon/nitrogen conditions in the WWTPs, as were the members of Bdellovibrionota and Myxococcota described in the main text.

The second most abundant phylum was Bacteroidota, which included *Saprospiraceae* (7.31 %). Kondrotaite *et al*. (2022) reported that uncultured *Saprospiraceae* (e.g., OLB8 clade) are commonly observed in WWTPs based on global microbial community surveys and play potential roles in nutrient removal and macromolecule degradation based on metagenomic information (Kondrotaite et al., 2022). The third most abundant phylum was Chloroflexi, comprising *Caldilineaceae* (4.16 %), *Anaerolineaceae* (3.17 %), and uncultured clades A4b (2.08 %) and 1-20 (1.71 %). Although type strains of *Caldilineaceae* and *Anaerolineaceae* have been isolated from thermophilic environments (Sekiguchi et al., 2003), mesophilic filamentous bacteria associated with Chloroflexi are frequently observed in aerobic wastewater treatment ecosystems and are thought to be the causative agents of sludge bulking (Nierychlo et al., 2019; Speirs et al., 2019). *Intrasporangiaceae* containing *Tetraspharea* (Jiang et al.,

2016) and *Thiotrichaceae* containing *Thiothrix* (Nielsen et al., 2000) were detected in sludge bulking in the shared microbial constituents. Such filamentous bulking-associated populations, including *Anaerolineaceae*, *Caldilineaceae*, and *Intrasporangiaceae,* were positively correlated with TC. Although the mechanisms underlying sludge bulking may be complex, high carbon content is likely one of the causes of sludge bulking in WWTPs.

In addition to these three major phyla, many microorganisms related to wastewater treatment ecosystems have been identified as shared microbial constituents. Kristensen *et al*. suggested that members of the *Blastocatellaceae* of the Acidobacteria could potentially be polyphosphate-accumulating organisms (PAOs) based on metagenomic information (Kristensen et al., 2021). According to the FISH visualization of ammonium-oxidizing cultures inoculated with activated sludge samples, *Deinococcaceae*-related cells are located around *Nitrosomonas* organisms, implying their importance in the ammonia oxidation process (Tan et al., 2008). Recently, Myxococcota and Bdellovibrionota populations were reported to behave as active predators in activated sludge ecosystems (Zhang et al., 2023b). Within the uncultivated phyla *Ca*. Patescibacteria, *Ca*. Saccharimonadia, *Ca*. Parcubacteria (*Ca*. Paceibacteria), and *Ca*. Gracilibacteria were detected in the shared microbial constituents. In the main text, the genomic traits of the Myxococcota, Bdellovibrionota, and *Ca*. Patescibacteria have been described based on their predatory/parasitic interactions. The other microbial taxa included Firmicutes, Planctomycetota, Verrucomicrobiota, and *Ca*. Eremiobacterota have been observed in waste/wastewater-associated environments (de Celis et al., 2020; Ji et al., 2021; Juretschko et al., 2002; Parks et al., 2017); however, their eco-physiological roles in activated sludge remain unclear.

The ecological information on shared genomes within industrial and municipal WWTPs

Of the 1,184 metagenome-assembled bins belonging to 277 families, 180 bins were reconstructed from the activated sludge samples of municipal WWTP (process E1) (Table S6). Interestingly, 167 of the 180 bins (92.7 %) from E1 were belonging to 58 families and overlapped with the families observed in the bins from the industrial WWTPs. This result reflected the principal coordinate analysis, which showed no clear differences in microbial community composition between the municipal and industrial WWTPs (Fig. S1). 130 bins from E1 were identified as shared microbial constituents, distributing 36

families of the phyla Actinobacteriota, Bacteroidota, Bdellovibrionota, Gemmatimonadota, Myxococcota, NB1-j, Nitrospirota, Patescibacteria, Planctomycetota, Proteobacteria, Verrucomicrobiota, and WPS-2.

Phylogenetic preference in the predatory and parasitic bacteria were observed in the bins from E1 (Table S6). As for the phylum Bdellovibrionota, only one bin of the family *Bdellovibrionaceae* (E1_bin.532) was recovered from E1. Within the Myxococcota-related bins, 9 out of 11 bins of uncultured clade mle1-27 were derived from E1. The metagenomic bins of the families *Bdellovibrionaceae* and *Myxococcaceae*, which are well-known predatory bacterial linages, with potential predatory functions encoded in their genomes were recovered from all and six (except for D1) WWTPs, respectively (Fig. 3 and Table S6). Within the family *Bdellovibrionaceae*, there are genera *Bdellovibrio* (A1_bin.263) and *Pseudobdellovibrio* (A1_bin.159), which are known to be obligate predators (Koval et al., 2013; Rendulic et al., 2004; Waite et al., 2020). In addition, bins of the family *Bdellovibrionaceae* include several unclassified genera, such as PSRN01 (B1_bin.97), Ga0074137 (B2_bin.31 and B2_bin.546), and UBA2316 (D1_bin.7), whose functions have not been elucidated due to the lack of cultures/isolates. Through the prediction of potential predatory functions of Bdellovibrionota, this study discovered that the shared microbial constituent of the family *Bdellovibrionaceae* commonly possess the predatory-related gene arrays similar with known obligate predators, suggesting that members of the family *Bdellovibrionaceae* play as obligate predators in the WWTPs treating various types of wastewaters.

A bin of the genus *Archangium*_A (A1_bin.100), which is known as a facultative predator (Zhang et al., 2023a) as similar to the genus *Myxococcus* (Thiery and Kaimer, 2020) was found in the family *Myxococcaceae*. In addition to the family *Myxococcaceae*, five *Nannocystis* (B2_bin.23, D1_bin.104, D1_bin.75, D1_bin.90, and E1_bin.459), three *Labilithrix* (B1_bin.169, B1_bin.289, and B2_bin.117), two *Minicystis* (A1_bin.23 and E1_bin.463), four *Polyangium* (B1_bin.244, C1_bin.23, D1_bin.170, D1_bin.251), and one *Sandaracinus* (B2_bin.298) bins were detected from WWTPs treating various wastewater. Although the metabolic functions of these known genera of the Myxococcota were diverse (Murphy et al., 2021), members of Myxococcota are mostly facultative predators and are widely distributed in the activated sludge systems treating municipal wastewater (Zhang et al., 2023b). This study found that the metagenomic bins of the family *Myxococcaceae* with potential cell contact-dependent predatory functions were

successfully reconstructed from WWTPs treating fermentation and municipal wastewater, and also suggested that the predation mechanism of other Myxococcota members might be different from those of the *Myxococcaceae* according to the genome analysis (Fig. 4A). Considering the similar trends in negative correlation with TN and/or TC for the *Bdellovibrionaceae*, *Myxococcaceae*, *Haliangiaceae, Nannocystaceae, Phaselicystidaceae, Polyangiaceae,* and *Sandaracinaceae* (Fig. 2), obligate (i.e., *Bdellovibrionaceae*) and facultative (i.e., *Myxococcaceae* and other Myxococcota) predators likely coexist in the WWTPs treating fermentation, chemical, and municipal wastewater.

Three bins of *Ca.* Patescibacteria from E1 were classified only into the order *Ca*. Peribacterales [also known as order *Ca*. Absconditabacteriales (SR1) in the SILVA taxonomy] of the class *Ca*. Gracilibacteria. These observations suggested that elucidating the potential functions of predatory and parasitic bacteria in both industrial and municipal WWTPs is important for understanding the mechanisms of microbial predation and parasitism in activated sludge ecosystem (see Results and Discussion in the main text).

REFERENCES

Camacho, C., Coulouris, G., Avagyan, V., Ma, N., Papadopoulos, J., Bealer, K. and Madden, T.L. 2009. BLAST plus : architecture and applications. BMC Bioinformatics 10, 421.

Chaumeil, P.A., Mussig, A.J., Hugenholtz, P. and Parks, D.H. 2020. GTDB-Tk: a toolkit to classify genomes with the Genome Taxonomy Database. Bioinformatics 36(6), 1925-1927.

Davidov, Y., Huchon, D., Koval, S.F. and Jurkevitch, E. 2006. A new alpha-proteobacterial clade of Bdellovibrio-like predators: implications for the mitochondrial endosymbiotic theory. Environ Microbiol 8(12), 2179-2188.

Davison, H.R., Hurst, G.D.D. and Siozios, S. 2023. 'Candidatus Megaira' are diverse symbionts of algae and ciliates with the potential for defensive symbiosis. Microb Genom 9(3), mgen000950.

de Celis, M., Belda, I., Ortiz-Alvarez, R., Arregui, L., Marquina, D., Serrano, S. and Santos, A. 2020. Tuning up microbiome analysis to monitor WWTPs' biological reactors functioning. Sci Rep 10(1), 4079.

Dueholm, M.S., Nierychlo, M., Andersen, K.S., Rudkjobing, V., Knutsson, S., Albertsen, M., Nielsen, P.H. and Consortium, M.G. 2022. MiDAS 4: A global catalogue of full-length 16S rRNA gene sequences and taxonomy for studies of bacterial communities in wastewater treatment plants. Nat Commun 13(1), 1908.

Hahn, M.W., Schmidt, J., Koll, U., Rohde, M., Verbarg, S., Pitt, A., Nakai, R., Naganuma, T. and Lang, E. 2017. Silvanigrella aquatica gen. nov., sp nov., isolated from a freshwater lake, description of Silvanigrellaceae fam. nov and Silvanigrellales ord. nov., reclassification of the order Bdellovibrionales in the class Oligoflexia, reclassification of the families Bacteriovoracaceae and Halobacteriovoraceae in the new order Bacteriovoracales ord. nov., and reclassification of the family Pseudobacteriovoracaceae in the order Oligoflexales. Int J Syst Evol Microbiol 67(8), 2555-2568.

Ji, M.K., Williams, T.J., Montgomery, K., Wong, H.L., Zaugg, J., Berengut, J.F., Bissett, A., Chuvochina, M., Hugenholtz, P. and Ferrari, B.C. 2021. Candidatus Eremiobacterota, a metabolically and phylogenetically diverse terrestrial phylum with acid-tolerant adaptations. ISME J 15(9), 2692-2707.

Jiang, X.T., Guo, F. and Zhang, T. 2016. Population dynamics of bulking and foaming bacteria in a full-scale wastewater treatment plant over five years. Sci Rep 6, 24180.

Juretschko, S., Loy, A., Lehner, A. and Wagner, M. 2002. The microbial community composition of a nitrifying-denitrifying activated sludge from an industrial sewage treatment plant analyzed by the full-cycle rRNA approach. Syst Appl Microbiol 25(1), 84-99.

Kondrotaite, Z., Valk, L.C., Petriglieri, F., Singleton, C., Nierychlo, M., Dueholm, M.K.D. and Nielsen, P.H. 2022. Diversity and ecophysiology of the genus OLB8 and other abundant uncultured Saprospiraceae genera in global wastewater treatment systems. Front Microbiol 13, 917553.

Koval, S.F., Hynes, S.H., Flannagan, R.S., Pasternak, Z., Davidov, Y. and Jurkevitch, E. 2013. Bdellovibrio exovorus sp. nov., a novel predator of Caulobacter crescentus. Int J Syst Evol Microbiol 63(Pt 1), 146-151.

Kristensen, J.M., Singleton, C., Clegg, L.A., Petriglieri, F. and Nielsen, P.H. 2021. High diversity and fnctional potential of undescribed "Acidobacteriota" in Danish wastewater treatment plants. Front Microbiol 12, 643950.

Lambina, V.A., Afinogenova, A.V., Romai Penabad, S., Konovalova, S.M. and Pushkareva, A.P. 1982. [Micavibrio admirandus gen. et sp. nov]. Mikrobiologiia 51(1), 114-117.

Moncadas, L.S., Shabarova, T., Kavagutti, V.S., Bulzu, P.A., Chiriac, M.C., Park, S., Mukherjee, I., Ghai, R. and

Andrei, A.S. 2023. Rickettsiales' deep evolutionary history sheds light on the emergence of intracellular lifestyles. bioRxiv, doi: 10.1101/2023.1101.1131.526412

Murphy, C.L., Yang, R., Decker, T., Cavalliere, C., Andreev, V., Bircher, N., Cornell, J., Dohmen, R., Pratt, C.J., Grinnell, A., Higgs, J., Jett, C., Gillett, E., Khadka, R., Mares, S., Meili, C., Liu, J., Mukhtar, H., Elshahed, M.S. and Youssef, N.H. 2021. Genomes of Novel Myxococcota Reveal Severely Curtailed Machineries for Predation and Cellular Differentiation. Appl Environ Microbiol 87(23), e0170621.

Nielsen, P.H., de Muro, M.A. and Nielsen, J.L. 2000. Studies on the in situ physiology of *Thiothrix* spp. present in activated sludge. Environ Microbiol 2(4), 389-398.

Nierychlo, M., Andersen, K.S., Xu, Y.J., Green, N., Jiang, C.J., Albertsen, M., Dueholm, M.S. and Nielsen, P.H. 2020. MiDAS 3: An ecosystem-specific reference database, taxonomy and knowledge platform for activated sludge and anaerobic digesters reveals species-level microbiome composition of activated sludge. Water Res 182, 115955.

Nierychlo, M., Milobedzka, A., Petriglieri, F., McIlroy, B., Nielsen, P.H. and McIlroy, S.J. 2019. The morphology and metabolic potential of the Chloroflexi in full-scale activated sludge wastewater treatment plants. FEMS Microbiol Ecol 95(2), doi: 10.1093/femsec/fiy228.

Parks, D.H., Rinke, C., Chuvochina, M., Chaumeil, P.A., Woodcroft, B.J., Evans, P.N., Hugenholtz, P. and Tyson, G.W. 2017. Recovery of nearly 8,000 metagenome-assembled genomes substantially expands the tree of life. Nat Microbiol 2(11), 1533-1542.

Pasternak, Z., Pietrokovski, S., Rotem, O., Gophna, U., Lurie-Weinberger, M.N. and Jurkevitch, E. 2013. By their genes ye shall know them: genomic signatures of predatory bacteria. ISME J 7(4), 756-769.

Quast, C., Pruesse, E., Yilmaz, P., Gerken, J., Schweer, T., Yarza, P., Peplies, J. and Glockner, F.O. 2013. The SILVA ribosomal RNA gene database project: improved data processing and web-based tools. Nucleic Acids Res 41(D1), D590-D596.

Schrallhammer, M., Ferrantini, F., Vannini, C., Galati, S., Schweikert, M., Gortz, H.D., Verni, F. and Petroni, G. 2013. '*Candidatus* Megaira polyxenophila' gen. nov., sp nov.: Considerations on evolutionary history, host range and shift of early divergent Rickettsiae. PLoS One 8(8), e72581.

Sekiguchi, Y., Yamada, T., Hanada, S., Ohashi, A., Harada, H. and Kamagata, Y. 2003. *Anaerolinea thermophila* gen. nov., sp nov and *Caldilinea aerophila* gen. nov., sp nov., novel filamentous thermophiles that represent a previously uncultured lineage of the domain *Bacteria* at the subphylum level. Int J Syst Evol Microbiol 53(6), 1843-1851.

Speirs, L.B.M., Rice, D.T.F., Petrovski, S. and Seviour, R.J. 2019. The phylogeny, biodiversity, and ecology of the *Chloroflexi* in activated sludge. Front Microbiol 10, 2015.

Tan, N.C.G., Kampschreur, M.J., Wanders, W., van der Pol, W.L.J., van de Vossenberg, J., Kleerebezem, R., van Loosdrecht, M.C.M. and Jetten, M.S.M. 2008. Physiological and phylogenetic study of an ammonium-oxidizing culture at high nitrite concentrations. Syst Appl Microbiol 31(2), 114-125.

Waite, D.W., Chuvochina, M., Pelikan, C., Parks, D.H., Yilmaz, P., Wagner, M., Loy, A., Naganuma, T., Nakai, R., Whitman, W.B., Hahn, M.W., Kuever, J. and Hugenholtz, P. 2020. Proposal to reclassify the proteobacterial classes Deltaproteobacteria and Oligoflexia, and the phylum Thermodesulfobacteria into four phyla reflecting major functional capabilities. Int J Syst Evol Microbiol 70(11), 5972-6016.

Zhang, L., Dong, C., Wang, J., Liu, M., Wang, J., Hu, J., Liu, L., Liu, X., Xia, C., Zhong, L., Zhao, Y., Ye, X., Huang, Y., Fan, J., Cao, H., Wang, J., Li, Y., Wall, D., Li, Z. and Cui, Z. 2023a. Predation of oomycetes by myxobacteria via a specialized CAZyme system arising from adaptive evolution. ISME J 17(7), 1089-1103.

Zhang, L., Huang, X.Y., Zhou, J.Z. and Ju, F. 2023b. Active predation, phylogenetic diversity, and global

prevalence of myxobacteria in wastewater treatment plants. ISME J, 17(5):671-681.
